# Immunomodulatory and Antidiabetic Effects of a New Herbal Preparation (HemoHIM) on Streptozotocin-Induced Diabetic Mice

**DOI:** 10.1155/2014/461685

**Published:** 2014-06-18

**Authors:** Jong-Jin Kim, Jina Choi, Mi-Kyung Lee, Kyung-Yun Kang, Man-Jeong Paik, Sung-Kee Jo, Uhee Jung, Hae-Ran Park, Sung-Tae Yee

**Affiliations:** ^1^Department of Biology, Sunchon National University, 255 Joongang-Ro, Seokhyeon-Dong, Suncheon 549-742, Republic of Korea; ^2^Department of Food and Nutrition, Sunchon National University, Suncheon, Republic of Korea; ^3^Department of Pharmacy, Sunchon National University, 255 Joongang-Ro, Seokhyeon-Dong, Suncheon 549-742, Republic of Korea; ^4^Radiation Research Division for Bio-Technology, Advanced Radiation Technology Institute, Jeongeup Campus of Korea Atomic Energy Research Institute (KAERI), Jeongeup, Republic of Korea

## Abstract

HemoHIM (a new herbal preparation of three edible herbs: *Angelica gigas* Nakai, *Cnidium officinale* Makino, and *Paeonia japonica* Miyabe) was developed to protect immune, hematopoietic, and self-renewal tissues against radiation. This study determined whether or not HemoHIM could alter hyperglycemia and the immune response in diabetic mice. Both nondiabetic and diabetic mice were orally administered HemoHIM (100 mg/kg) once a day for 4 weeks. Diabetes was induced by single injection of streptozotocin (STZ, 200 mg/kg, i.p.). In diabetic mice, HemoHIM effectively improved hyperglycemia and glucose tolerance compared to the diabetic control group as well as elevated plasma insulin levels with preservation of insulin staining in pancreatic *β*-cells. HemoHIM treatment restored thymus weight, white blood cells, lymphocyte numbers, and splenic lymphocyte populations (CD4^+^ T and CD8^+^ T), which were reduced in diabetic mice, as well as IFN-*γ* production in response to Con A stimulation. These results indicate that HemoHIM may have potential as a glucose-lowering and immunomodulatory agent by enhancing the immune function of pancreatic *β*-cells in STZ-induced diabetic mice.

## 1. Introduction

Diabetes mellitus (DM) is one of the leading causes of morbidity and mortality worldwide [1]. Increases in the aging population, consumption of calorie-rich diets, obesity, and sedentary lifestyle have led to a tremendous surge in the number of diabetics [2]. Likewise, incidence of diabetes in Korea has increased rapidly in the past 10 years, becoming the 4th leading cause of death [3].

Type 1 DM results from the selective destruction of insulin-producing *β*-cells in pancreatic islets, and it is primarily a T cell-mediated autoimmune disease directed against one or more *β*-cell autoantigens [4, 5]. This state is characterized by limited weight gain, polyuria, polydipsia, and polyphagia attributed to the decreased capability of insulin to stimulate glucose uptake and utilization in target tissues due to insulin resistance, insulin insufficiency, and changes in other factors such as glucagon, thyroxin, glucocorticoid, and catecholamines [1].

A number of studies on oral antihyperglycemic agents derived from plants used in traditional Oriental medicine have been conducted, and many of the plants were found to have good activity [6]. The World Health Organization (WHO) has also recommended the evaluation of plants' effectiveness whenever safe modern drugs are unavailable [7]. This has led to an increased demand for research on natural products with antidiabetic activity as well as minimal to no side effects [8]. Unfortunately, complete therapy for DM and its complications has not been established yet.

In traditional Oriental medicine, many herbs or herbal prescriptions comprising several medicinal plants have been reputed to promote health, improve the defense mechanisms of the body, and enhance longevity [9]. A new herbal preparation (HemoHIM) was designed to protect self-renewing tissues as well as promote recovery of the immune system [10]. HemoHIM was first prepared by adding its polysaccharide fraction to the hot water extract of a herbal mixture consisting of Angelica Radix, Cnidii Rhizoma, and Paeonia Radix [11]. In our previous study, HemoHIM rescued white blood cells, and lymphocytes were reduced using ionizing radiation (IR). Also, we previously observed that HemoHIM was effective for the restoration of impaired immune functions [9] and its anti-inflammatory activity against carrageenan-induced edema, the formation of granulation tissues by cotton pellet, and experimental colitis by 2,4,6-trinitrobenzene sulfonic acid [11]. In addition, HemoHIM enhances the therapeutic efficacy in tumor-bearing mice treated with a chemotherapeutic agent or IR [12, 13]. Overall, these data suggest that HemoHIM can act as a potential modifier of biological responses. The general composition of HemoHIM is 60.4% carbohydrate, 6% protein, and 33.6% other including polyphenols (data not shown). The immune modulating components in HemoHIM were the ethanol-insoluble fraction [14, 15], the polysaccharide content of which was 40.9% (±3.8) (data not shown). Other carbohydrate components in HemoHIM were acidic-polysaccharide ethanol-soluble fraction, the polysaccharide content of which was 19.5% (data not shown). In addition, the functional components included in the ethanol-soluble fraction of HemoHIM were gallic acid [0.2% (±0.06)], chlorogenic acid [0.33% (±0.05)], paeoniflorin [1.32% (±0.15)], nodakenin [0.58% (±0.04)], and benzoic acid [0.17% (±0.05)]. In particular, these herbs are listed as raw materials in the Korean Food Code. Finally, HemoHIM has been proven to be safe for long-term administration [16, 17].

Streptozotocin (STZ) is 1-methyl-1-nitrosourea derivative of 2-deoxy-Dglucose and a broad antibacterial agent that can cause symptoms of diabetes. Various studies reported antidiabetic effects using STZ-induced diabetic mice model [18, 19]. In the current study, we investigated whether or not HemoHIM could improve immune function and antidiabetic effect on STZ-induced diabetic mice.

## 2. Materials and Methods

### 2.1. Preparation of HemoHIM

A mixture of three edible medicinal herbs, Angelica Radix (root of* Angelica giga*s Nakai), Cnidii Rhizoma (rhizome of* Cnidium officinale* Makino), and Paeonia Radix (root of* Paeonia japonica* Miyabe) was decocted for 4 h in boiling water to obtain a total extract. A part of the total extract was fractionated into an ethanol-soluble fraction and an ethanol-insoluble polysaccharide fraction by precipitation in 80% ethanol. HemoHIM was prepared by adding the polysaccharide fraction to the other part of the total extract [9].

### 2.2. Experimental Animals

Six-week-old male ICR mice were purchased from Orient Inc. (Charles River Technology, Seoul, Korea). The mice were individually housed and maintained at a controlled temperature (22 ± 2°C) and relative humidity (50 ± 5%) under a 12 h light-dark cycle. All mice were fed a pelletized commercial chow diet for 7 days after arrival. Next, the animals were randomly divided into four groups of 10 mice each: nondiabetic control group (nondiabetic), nondiabetic group administered HemoHIM (nondiabetic + HemoHIM), diabetic control group (diabetic), and diabetic group administered HemoHIM (diabetic + HemoHIM). The mice had access to food and water* ad libitum*. Both nondiabetic and diabetic mice were orally administered HemoHIM (100 mg/kg) once a day for 4 weeks. Body weights of mice were measured weekly. All mice were treated in strict accordance with Sunchon National University Institutional Animal Care and Use Committee (SCNU IACUC) guidelines for the care and use of laboratory animals. All procedures were approved by the SCNU IACUC.

### 2.3. Induction of Diabetes in Mice

Diabetes was induced by a single injection of STZ (200 mg/kg body weight) (Sigma, St. Louis, MO, USA) freshly dissolved in 0.1 M citrate buffer (pH 4.2) into the intraperitoneal injection. Nondiabetic mice were injected with citrate buffer alone. After 48 h, only STZ-treated mice that exhibited a fasting blood glucose level ≥200 mg/dL were used in the study.

### 2.4. Fasting Blood Glucose Levels and Oral Glucose Tolerance Test (OGTT)

Blood glucose levels were monitored in venous blood drawn from the tail using a glucometer (ACCU-CHEK, Roche, Germany) every week after 12 h of fasting. The OGTT was performed in the 4th week. Following 12 h of fasting, the mice were orally administered glucose at 1 g/kg of body weight, after which blood glucose levels were measured from the tail vein at 30, 60, and 120 min after glucose administration.

### 2.5. Plasma Insulin Levels

Blood was collected in a heparin-coated tube and centrifuged at 3,000 rpm for 5 min at 4°C. The plasma insulin levels were determined using a quantitative sandwich enzyme immunoassay kit (ELISA kit, Shibayagi Co., Ltd., Japan).

### 2.6. Immunohistochemistry of Pancreas

To measure insulin levels, immunohistochemical analysis was performed using a mouse anti-insulin monoclonal antibody (BioGenex, Netherlands). Briefly, paraffin sections of 4 *μ*m thickness were treated with 3% H_2_O_2_ in methanol for 30 min to block any endogenous peroxidase, followed by washing with 0.01 M phosphate buffer for 10 min. Next, the sections were processed by an indirect immunoperoxidase technique using a commercial kit (ABC kit, DAKO, USA) with secondary antibodies. The sections were then counterstained with Mayer's hematoxylin, and the staining was visualized by incubation with diaminobenzidine (DAB) (Zymed Laboratories, San Francisco, CA, USA) and counterstaining with 1% methyl green for 1 min [20].

### 2.7. Plasma Hematological Change and Biomarkers

Hematological change analysis was carried out using an automatic analyzer (HEMAVET 850, USA) for white blood cells (WBCs) and lymphocytes. Serum GOT (glutamate oxaloacetate transaminase), GPT (glutamic pyruvic transaminase), LDH (lactate dehydrogenase), and ALP (alkaline phosphatase) activities were determined using an automatic analyzer (FUJI DRI-CHEM 3500, Japan).

### 2.8. Cell Surface Markers Assay

Spleen cells (1 × 10^6^ cells/mL) were blocked with anti-CD16/32 (Fc*γ*II/III receptor) mAb for 30 min at 4°C and then washed with PBS solution containing 1% FBS and 0.1% NaN_3_. The cells were stained with PE-conjugated anti-CD8 mAb and FITC-conjugated anti-CD4 mAb or FITC-conjugated anti-CD19 mAb for 30 min at 4°C. Stained cells were then washed and detected by a flow cytometer (COULTER Epics XL, USA).

### 2.9. Cytokines and Immunoglobulins Analysis

Spleen cells (5 × 10^6^ cells/mL) were treated with Con A (1 *μ*g/mL) or LPS (10 *μ*g/mL) for 24 or 48 h, after which culture supernatants were harvested and stored at −20°C until use. Cytokine and immunoglobulin contents were measured by an enzyme-linked immunosorbent assay kit (ELISA) (Pharmigen, San Diego, CA, USA). All samples were tested in triplicate in accordance with standard curves. The sensitivity of each assay was as follows: IL-2, IFN-*γ*, IL-6, and TNF-*α*, 10 pg/mL; IgM and IgG1, 100 pg/mL.

### 2.10. Statistical Analysis

All data are presented as the mean ± SD. Statistical analyses were performed using the SPSS program (SPSS, Chicago, IL). Student's* t*-test was used to assess the differences between the groups. The nondiabetic group was compared with the nondiabetic + HemoHIM, diabetic, and diabetic + HemoHIM groups. The effects of HemoHIM administration were also compared within diabetic mice groups. Values of *P* < 0.05 were considered to be statistically significant.

## 3. Results

### 3.1. Body Weight and Organ Weight Changes

**Figure 1:**
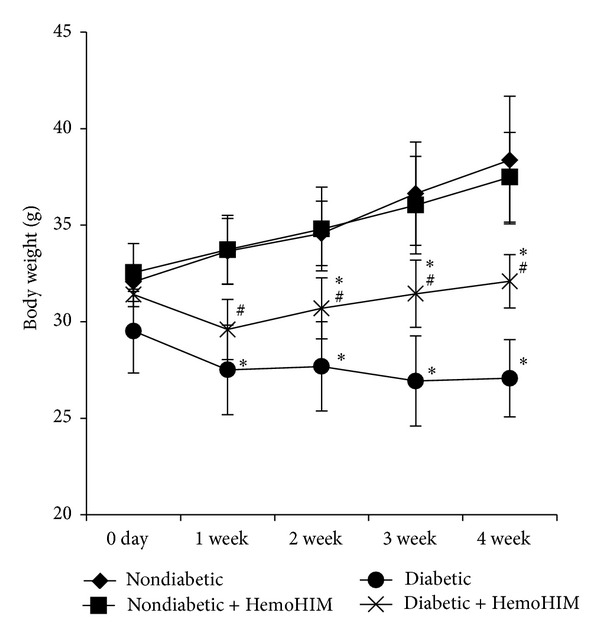
Effects of HemoHIM on changes in body weight of STZ-induced diabetic mice. ICR mice orally administrated HemoHIM (4 weeks) before being injected with STZ. Mice body weights were measured weekly. Values are expressed as the mean ± S.D. *P < 0.05 compared with nondiabetic group. ^#^ *P* < 0.05 compared with diabetic group.

During the experimental period, the body weight of diabetic mice was significantly lower than that of nondiabetic mice. However, HemoHIM administration suppressed the reduction of body weight due to diabetes from the 1st week in diabetic mice (Figure 1).

Relative weights of the liver, kidney, and lung were significantly higher in diabetic control mice compared to nondiabetic control mice, whereas thymus weight was lower. Although HemoHIM administration did not affect the organ weights of nondiabetic mice, it effectively recovered those of diabetic mice (Table 1).

### 3.2. Blood Glucose Level and Oral Glucose Tolerance Test (OGTT)

**Figure 2:**
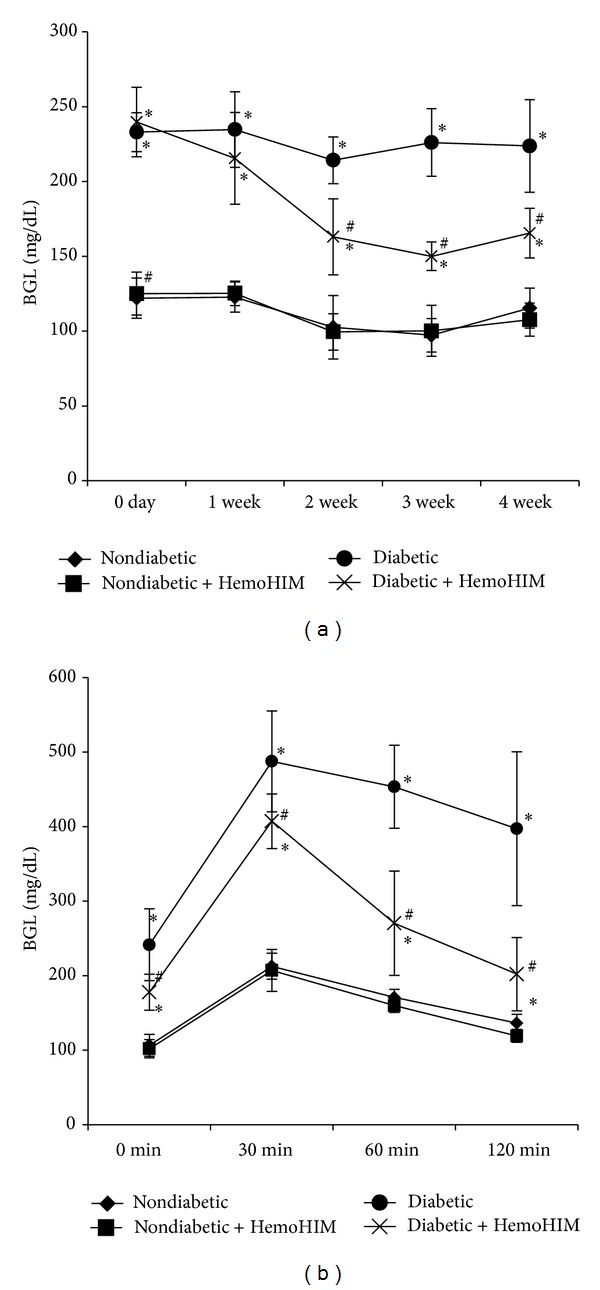
ICR mice orally administrated HemoHIM (4 weeks) before being injected with STZ. Blood glucose levels were monitored in venous blood drawn from the tail (a). The oral glucose tolerance test was performed in the 4th week. Following 12 h of fasting, the mice were orally administered glucose at 1 g/kg of body weight, after which blood glucose levels were measured from the tail vein at 30, 60, and 120 min after glucose administration (b). Values are expressed as means ± S.D. *P < 0.05 compared with nondiabetic group. ^#^ *P* < 0.05 compared with diabetic group.

HemoHIM administration did not affect blood glucose levels in nondiabetic mice. However, it significantly reduced blood glucose levels in diabetic mice treated with HemoHIM compared to the diabetic control group from the 2nd to 4th week. At the end of experiment, HemoHIM administration had significantly reduced the fasting blood glucose level by 26% in diabetic mice (Figure 2(a)).

To determine the effects of HemoHIM on the postprandial glucose level, we performed an oral glucose tolerance test. Blood glucose reached its highest level at 30 min after glucose administration. Blood glucose levels of the diabetic group treated with HemoHIM were significantly lower than those of the diabetic control group (Figure 2(b)). Thus, HemoHIM effectively improved fasting glucose and postprandial blood glucose levels in STZ-induced diabetic mice.

### 3.3. Plasma Insulin Level and Pancreatic Immunohistochemistry

**Figure 3:**
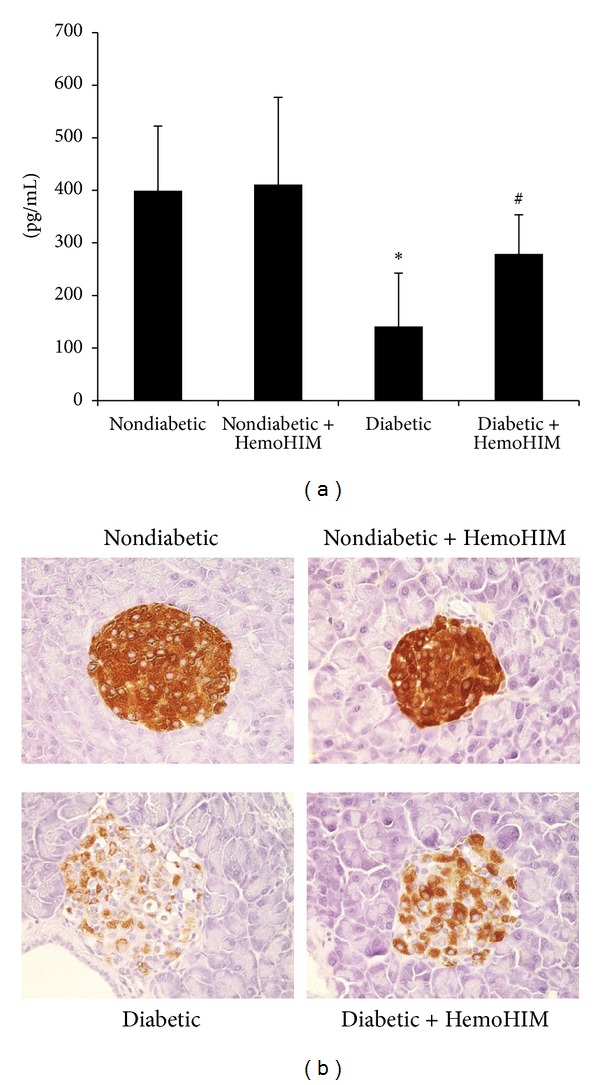
ICR mice orally administrated HemoHIM (4 weeks) before being injected with STZ. Effect of HemoHIM administration on plasma (a) and pancreatic *β*-cell ((b), ×200) insulin levels in STZ-induced diabetic mice.Pancreas was immunohistochemically stained as described in Materials and Methods. Values are expressed as means ± S.D. *P < 0.05 compared with nondiabetic group. ^#^ *P* < 0.05 compared with diabetic group.

There was no difference in the plasma insulin level between nondiabetic mice and nondiabetic mice administered HemoHIM. The plasma insulin level of the diabetic control group was significantly reduced compared to that of the nondiabetic group, whereas HemoHIM significantly increased the plasma insulin level by approximately 2-fold (Figure 3(a)).

Reduction of the *β*-cell population was evident in the diabetic control group compared to the nondiabetic group based on changes in the immunohistochemistry of the pancreas. In the nondiabetic group, insulin-positive *β*-cells were found throughout the islets, whereas only a few insulin-positive *β*-cells were sporadically scattered in the islets of the diabetic group. On the other hand, the insulin-positive *β*-cells in the diabetic group treated with HemoHIM showed strong staining and were preserved within distinct boundaries compared to those of the diabetic control group (Figure 3(b)).

### 3.4. Splenic Lymphocyte Subpopulations

**Figure 4:**
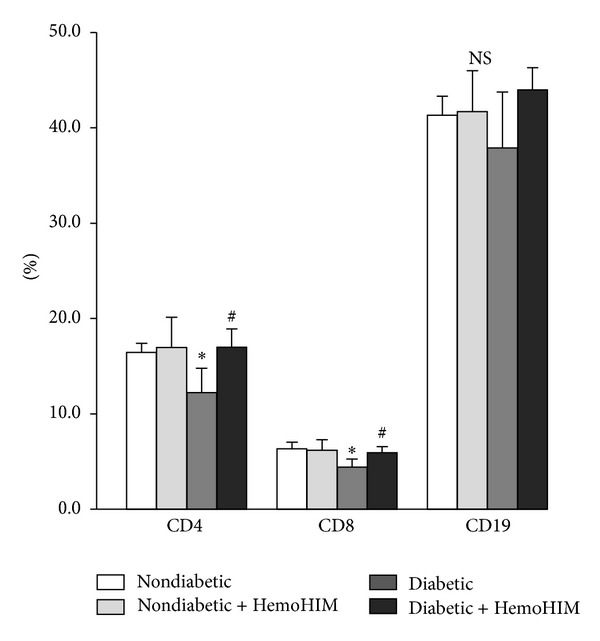
Effects of HemoHIM on splenocyte subpopulation in STZ-induced diabetic mice. Spleen cells (1 × 10^6^ cells/mL) were stained with PE-anti-CD8, FITC-anti-CD4, or FITC-anti-CD19 for 30 min at 4°C after blocking Fc*γ*II/III receptor. Stained cells were analyzed by a flow cytometer. Values are expressed as means ± S.D. *P < 0.05 compared with nondiabetic group. ^#^ *P* < 0.05 compared with diabetic group.

Splenic lymphocyte CD4^+^ T (helper T cell) and CD8^+^ T (cytotoxic T cell) cell populations were significantly reduced in diabetic control mice compared to nondiabetic mice (Figure 4). However, HemoHIM increased the number of CD4^+^ and CD8^+^ lymphocyte subpopulations compared to the diabetic control group. Counts of CD19^+^ cells were not different between the groups (Figure 4).

### 3.5. Production of Cytokine and Immunoglobulins by Splenocytes

The IFN-*γ* level of the diabetic group was reduced to about 56% of that of the nondiabetic group. On the other hand, the diabetic group treated with HemoHIM showed restored IFN-*γ* levels. The IL-6 level increased in the diabetic group, showing values significantly higher than those of the nondiabetic group, whereas the diabetic group treated with HemoHIM showed restored IL-6 levels (Table 2). After stimulation with LPS, immunoglobulins were measured in the supernatant of splenocytes. Levels of IgM and IgG1 were increased in the diabetic mice compared with the nondiabetic group. On the other hand, the diabetic group treated with HemoHIM showed reduced immunoglobulin secretion levels (Table 2).

### 3.6. Blood Hematological and Plasma Biomarker Changes

**Figure 5:**
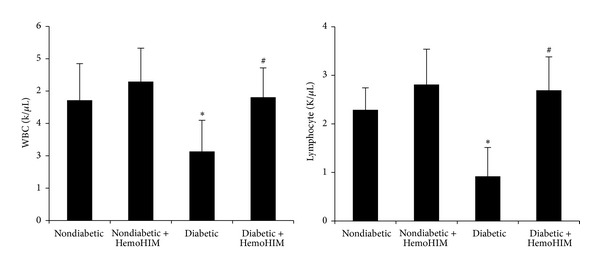
ICR mice orally administrated HemoHIM (4 weeks) before being injected with STZ. Hematological change analysis was carried out using an automatic analyzer (HEMAVET 850, USA) for white blood cells (WBCs) and lymphocytes. Values are expressed as means ± S.D. WBC: white blood cell. *P < 0.05 compared with nondiabetic group. ^#^ *P* < 0.05 compared with diabetic group.

Lymphocyte and WBC numbers were significantly reduced in the diabetic groups compared to nondiabetic groups. However, HemoHIM treatment recovered these numbers too close to nondiabetic values (Figure 5). Although serum GOT, GPT, LDH, and ALP activities were significantly higher in the diabetic group than in the nondiabetic group by 2.2-, 2.2, 1.7, and 2-fold, respectively, HemoHIM administration reduced them compared to the diabetic control group (Table 3).

## 4. Discussion 

This study was conducted to investigate the effects of a new herbal preparation (HemoHIM) on immune function and hyperglycemia in STZ-induced diabetic mice as a novel diabetic remedy. HemoHIM consists of three kinds of edible herbs,* Angelica gigas* Nakai,* Cnidium officinale* Makino, and* Paeonia japonica *Miyabe, which have been reported as possessing antidiabetic and antihyperglycemic activities [21–23].

Since STZ is known to cause selective destruction of *β*-cells within islets of Langerhans, resulting in marked reduction of insulin levels, it makes sense that glycogen levels in tissues decrease as they depend on insulin for influx of glucose [24–26]. HemoHIM administration significantly reduced fasting blood glucose levels while elevating plasma insulin levels in STZ-induced diabetic mice. The plasma insulin level of diabetic control mice was about 35% of that of nondiabetic mice. However, HemoHIM administration elevated the plasma insulin level by 200% compared to that of the diabetic control group. In this study, OGTT of diabetic mice was significantly higher than that in nondiabetic mice. However, HemoHIM administration effectively improved postprandial blood glucose levels. As such, these results suggest that constituents of HemoHIM may inhibit the increase in blood glucose levels due to enhancement of insulin secretion in STZ-induced diabetic mice. We also observed that HemoHIM effectively preserved pancreatic *β*-cells compared to the diabetic control group using immunohistochemistry assay. In addition, some studies reported that STZ-diabetic mice induce infiltration of lymphocytes to the pancreas [27]. To investigate the infiltration of lymphocytes, pancreas was stained with H&E (see Supplement Figure 1 available online at http://dx.doi.org/10.1155/2014/461685). This experiment revealed that lymphocytes infiltration was reduced by HemoHIM in the diabetic mice.

Diabetic mice exhibit hyperglycemia in the form of polydipsia, polyphagia, polyuria, and body weight loss [28]. The body weight of the diabetic control group decreased due to insulin deficiency and dysfunctional energy metabolism [5, 10]. During the experimental period, the nondiabetic mice showed an approximately 20% body weight gain, whereas the body weight of diabetic mice decreased by about 8% over the same time period. However, loss of body weight was suppressed by HemoHIM. Failure of STZ-induced diabetic animal models to gain body weight has already been reported [29, 30]. Kidney and liver weights are higher in diabetic animals, as STZ-induced diabetic animals undergo glomerular hypertrophy [31] and triglyceride accumulation [30] in the kidney and liver, respectively. In the current study, HemoHIM attenuated hypertrophy of organs (kidney, liver, and lung) and the thymus in STZ-induced diabetic mice [32, 33]. However, HemoHIM protected loss of kidney weight in the STZ-induced diabetic mice but did not affect creatinine (data not shown). Dağistanli et al. [34] reported that thymic atrophy is caused by elevation of intracellular calcium levels, leading to apoptosis in STZ-induced diabetes. We also found that the relative thymus weight was dramatically reduced by 44% in STZ-induced diabetic mice compared to the nondiabetic group. However, HemoHIM supplementation significantly improved thymic atrophy by 1.8-fold compared to the diabetic control group, which indicates that HemoHIM may protect the decline of immune response.

Furthermore, hyperglycemia is toxic to multiple immune cell populations, including lymphocytes [35]. Therefore, we determined whether or not HemoHIM affects splenocyte subpopulation in diabetic mice. Recently, immunological injury was reported to be associated with DM. Avanzini et al. [36] reported that IFN-*γ* is reduced in DM, as the percentage of peripheral CD4^+^ and CD8^+^ cells was found to be significantly lower in DM patients. The current study showed that spleen cells from the diabetic group produced significantly less IFN-*γ* in response to Con A. However, HemoHIM recovered IFN-*γ* production in STZ-induced diabetic mice. Further, the HemoHIM-treated diabetic group showed increased CD4^+^ and CD8^+^ cell numbers in the spleen. In aged mice and airway inflammation mice, HemoHIM has been shown to restore the Th1/Th2 balance [9, 37]. Various immunoglobulins are increased in DM [38, 39]. Specifically, IgM and IgG1 were increased in the diabetic group in the present study. On the other hand, immunoglobulin levels of the HemoHIM-treated diabetic group were reduced compared with the diabetic control group. These data suggest that HemoHIM treatment can overcome immunological injuries in diabetic mice. Thus, HemoHIM has potential immunomodulatory activity in DM. Further, GOT, GPT, LDH, and ALP were significantly increased in the diabetic group, whereas the diabetic group treated with HemoHIM showed restored levels. In addition, hematological results indicated that HemoHIM could reduce liver toxicity in STZ-induced diabetic mice.

In conclusion, HemoHIM improved the symptom of STZ-induced diabetic. The evidence of these characteristics of HemoHIM includes (1) restoration from destroyed *β*-cells by STZ; (2) increase in blood insulin level; (3) decrease in blood glucose level by increased insulin; (4) protection of body and organs weight from disruption by STZ; (5) inhibitition of immunological changes by STZ. On the basis of the results described herein, we suggest that HemoHIM has antidiabetic potential against increase in blood glucose levels and even immune system disruption.

## Supplementary Material

To investigate the infiltration of lymphocytes, pancreas was stained with H&E. This experiment revealed that lymphocytes infiltration was reduced by HemoHIM in the diabetic mice.

## Figures and Tables

**Table 1:** Changes in organ weights in mice administered HemoHIM.

	Nondiabetic	Nondiabetic + HemoHIM	Diabetic	Diabetic + HemoHIM
Liver (mg/g)	39.25 ± 2.74	38.93 ± 0.97	48.39 ± 3.45∗	42.07 ± 4.55^#^
Kidney (mg/g)	13.49 ± 0.23	13.44 ± 0.61	19.79 ± 1.01∗	15.22 ± 1.61^∗,#^
Thymus (mg/g)	1.30 ± 0.12	1.14 ± 0.11	0.58 ± 0.10∗	1.03 ± 0.14^∗,#^
Lung (mg/g)	4.84 ± 2.74	4.92 ± 0.10	6.06 ± 0.36∗	5.54 ± 0.26^∗,#^
Spleen (mg/g)	2.73 ± 0.19	2.96 ± 0.21	2.56 ± 0.41	2.77 ± 0.44
Heart (mg/g)	4.65 ± 0.22	4.74 ± 0.43	4.42 ± 0.11	4.41 ± 0.32

Values are expressed as means ± S.D.

*P < 0.05 compared with nondiabetic group.

^#^ *P* < 0.05 compared with diabetic group.

**Table 2:** Cytokine and immunoglobulin production by spleen cells in mice administered HemoHIM.

	Nondiabetic	Nondiabetic + HemoHIM	Diabetic	Diabetic + HemoHIM
Cytokines (ng/mL)				
IL-2	1.21 ± 0.22	1.21 ± 0.19	1.22 ± 0.29	1.22 ± 0.10
IFN-*γ*	6.61 ± 1.60^#^	6.74 ± 2.37^#^	2.85 ± 1.86∗	5.01 ± 2.39
IL-6	1.03 ± 0.28^#^	1.15 ± 0.30^#^	1.57 ± 0.28∗	0.90 ± 0.31^#^
TNF-*α*	0.39 ± 0.08	0.35 ± 0.15	0.53 ± 0.23	0.35 ± 0.08
Immunoglobulin (ng/mL)				
IgM	616.0 ± 163.2	586.4 ± 93.4^#^	873.5 ± 231.9	566.6 ± 290.6
IgG1	5.0 ± 2.7^#^	5.2 ± 2.7	9.7 ± 3.6∗	6.7 ± 1.2

The spleen cells (5 × 10^6^ cells/mL) were treated with Con A (1 *μ*g/mL) or LPS (10 *μ*g/mL) for 24 hours and culture supernatants were harvested and stored at −20°C until use. IL-2 and IFN-*γ* were measured in the Con A-stimulated supernatant. IL-6, TNF-*α*, IgM, and IgG1 were measured in the LPS-stimulated supernatant. Values are expressed as means ± S.D.

*P < 0.05 compared with nondiabetic group.

^#^ *P* < 0.05 compared with diabetic group.

**Table 3:** Plasma biomarkers in mice administered HemoHIM.

	Nondiabetic	Nondiabetic + HemoHIM	Diabetic	Diabetic + HemoHIM
GOT (U/L)	55 ± 7	50 ± 9	126 ± 27∗	73 ± 12^#^
GPT (U/L)	24 ± 6	21 ± 6	52 ± 23∗	28 ± 6
LDH (U/L)	368 ± 117	301 ± 31	618 ± 108∗	489 ± 112
ALP (U/L)	124 ± 11	120 ± 10	281 ± 56∗	193 ± 20^#^

GOT: glutamate oxaloacetate transaminase, GPT: glutamic pyruvic transaminase, LDH: lactate dehydrogenase, ALP: alkaline phosphatase. Values are expressed as means ± S.D.

*P < 0.05 compared with nondiabetic group.

^#^ *P* < 0.05 compared with diabetic group.
