# Supplementary material for: Immunomodulatory and Antidiabetic Effects of a New Herbal Preparation (HemoHIM) on Streptozotocin-Induced Diabetic Mice
Source: Evid Based Complement Alternat Med. 2014 Jun 18;2014:461685. doi: 10.1155/2014/461685 (PMC4087252; doi:10.1155/2014/461685)
Supplement: Supplementary file 1 — To investigate the infiltration of lymphocytes, pancreas was stained with H&E. This experiment revealed that lymphocytes infiltration was reduced by HemoHIM in the diabetic mice. [file 461685.f1.pdf]

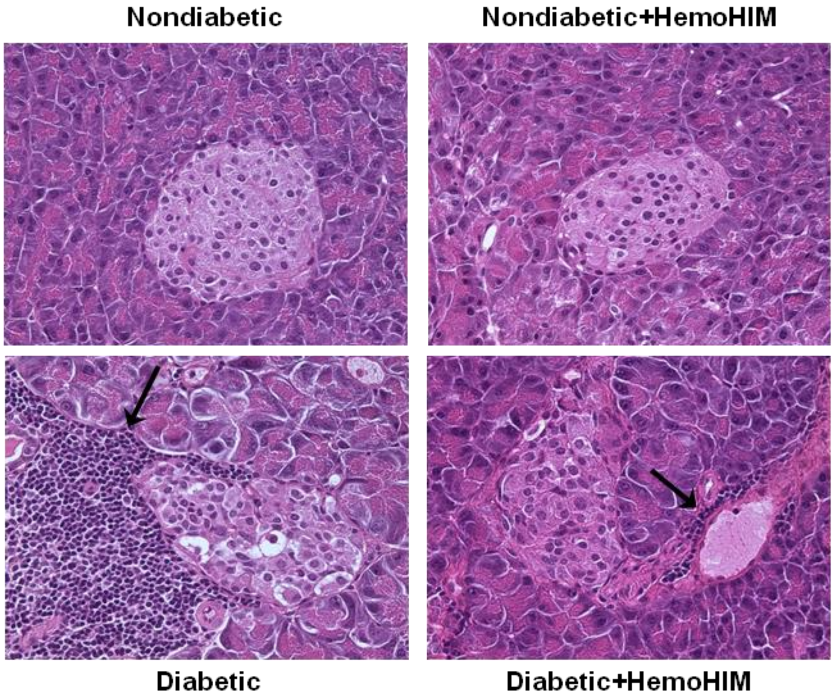

Supplement figure 1. Mouse pancreas was harvested and paraffin embedded after fixation in formalin. Four-micron sections were cut and stained with Hematoxylin & Eosin (H&E ×400). Nondiabetic and nondiabetic+HemoHIM mice were not detected infiltration of lymphocyte in the pancreas. Lymphocytes were infiltrated surrounding islet cells in the STZ-induced diabetic mice (black arrow). Interestingly, however, HemoHIM dramatically reduced infiltration of lymphocytes (black arrow).
